# Evolutionary engineering of a wine yeast strain revealed a key role of inositol and mannoprotein metabolism during low-temperature fermentation

**DOI:** 10.1186/s12864-015-1755-2

**Published:** 2015-07-22

**Authors:** María López-Malo, Estéfani García-Rios, Bruno Melgar, Monica R Sanchez, Maitreya J Dunham, José Manuel Guillamón

**Affiliations:** Departamento de Biotecnología de los alimentos, Instituto de Agroquímica y Tecnología de los Alimentos (CSIC), Avda. Agustín Escardino, 7, E-46980 Paterna, Valencia Spain; Department of Genome Sciences, University of Washington, Seattle, WA 98195 USA

**Keywords:** Wine, Yeast, Experimental evolution, Cold, Fermentation, Inositol

## Abstract

**Background:**

Wine produced at low temperature is often considered to improve sensory qualities. However, there are certain drawbacks to low temperature fermentations: e.g. low growth rate, long lag phase, and sluggish or stuck fermentations. Selection and development of new *Saccharomyces cerevisiae* strains well adapted at low temperature is interesting for future biotechnological applications. This study aimed to select and develop wine yeast strains that well adapt to ferment at low temperature through evolutionary engineering, and to decipher the process underlying the obtained phenotypes.

**Results:**

We used a pool of 27 commercial yeast strains and set up batch serial dilution experiments to mimic wine fermentation conditions at 12 °C. Evolutionary engineering was accomplished by using the natural yeast mutation rate and mutagenesis procedures. One strain (P5) outcompeted the others under both experimental conditions and was able to impose after 200 generations. The evolved strains showed improved growth and low-temperature fermentation performance compared to the ancestral strain. This improvement was acquired only under inositol limitation. The transcriptomic comparison between the evolved and parental strains showed the greatest up-regulation in four mannoprotein coding genes, which belong to the *DAN*/*TIR* family (*DAN1, TIR1, TIR4* and *TIR3*). Genome sequencing of the evolved strain revealed the presence of a SNP in the *GAA1* gene and the construction of a site-directed mutant (*GAA1*^*Thr108*^) in a derivative haploid of the ancestral strain resulted in improved fermentation performance. *GAA1* encodes a GPI transamidase complex subunit that adds GPI, which is required for inositol synthesis, to newly synthesized proteins, including mannoproteins.

**Conclusions:**

In this study we demonstrate the importance of inositol and mannoproteins in yeast adaptation at low temperature and the central role of the *GAA1* gene by linking both metabolisms.

**Electronic supplementary material:**

The online version of this article (doi:10.1186/s12864-015-1755-2) contains supplementary material, which is available to authorized users.

## Background

Low temperatures (10-15 °C) are used in wine fermentations to enhance production and to retain flavor volatiles. In this way, white and “rosé” wines of greater aromatic complexity can be achieved [1, 2]. However, lowering fermentation temperatures has its disadvantages, including prolonged process duration and a higher risk of halted or sluggish fermentation [3]. These problems can be avoided by providing better-adapted yeasts to ferment at low temperature. Although the wine industry already has yeasts that are sold as cryotolerant strains, most do not offer desirable fermentation performance at low temperature [4]. Thus, the selection of yeast able to ferment at low temperature is still of much interest for the winemaking industry [5–7]. Nevertheless, regarding low temperature fermentation, natural phenotypic diversity can be very limited in *S. cerevisiae* strains, the least psychrotrophic species of the *Saccharomyces* genus [8]. An appealing alternative is the development of genetically improved new strains of *S. cerevisiae* that are better adapted to grow at low temperature.

In recent decades, many efforts have been made to engineer wine yeast strains with improved characteristics [9–11]. However, metabolic engineering based on recombinant technology has its limitations: 1) requirement for extensive biochemical and genetic information of the metabolism of interest; 2) the complexity of the cellular physiological response, such as activation of an alternative metabolic pathway; 3) cloning difficulties in industrial strains, which result mainly from their genetic complexity; 4) regulatory issues such as using genetically modified organisms (GMO) in the food industry [12]. Nonrecombinant strategies based on evolutionary engineering are attractive because they may generate improved strains that are not considered GMOs, and will most likely be better accepted by the general public. Evolutionary engineering has been used for generating new industrial strains [13–15]. Bioethanol production is the most important area where this approach has been applied in yeast. However, very few studies have reported the development of improved wine yeast strains through evolutionary engineering [16–18].

Experiments for many generations, under conditions to which yeasts are not optimally adapted, help select for more fit genetic variants. Culturing *S. cerevisiae* populations under long-term selective pressures results in a series of adaptive shifts. These shifts have been observed to occur on the order of once in every 50 generations [19]. The initial (physical or chemical) mutagenesis of the starting culture potentiates increased genetic diversity [20]. Such experiments have also shed light on a bigger question about the molecular basis underlying the improved phenotype. Evolutionary engineering provides the opportunity to study evolutionary adaptation by analyzing either changes in gene expression patterns following adaptive evolution in yeast, or the genome structure and organization or the whole genome sequence of the evolved strains [21, 22, 19].

The first aim of this study was to assess the most competitive strains that grow under wine fermentation conditions at low temperature. To this end, we performed a growth competition assay with 27 commercial wine strains inoculated at equal population size in synthetic grape must. In spite of the economical and industrial importance of these strains, their phenotypic variation in the main enological traits, particularly those related to optimum growth temperature [8], and their ability to adapt to low temperature fermentation have been poorly investigated. The second goal was to obtain an improved strain to grow and ferment at low temperature by evolutionary engineering. For this purpose, we maintained growth competition in synthetic grape must during 200 generations to select for the mutations that produce phenotypes with improved growth in this medium. One of these evolved cultures was previously treated with ethyl methanesulfonate (EMS) to increase the mutation rate. Finally, we aimed to decipher the molecular basis underlying this improvement by analyzing the genomic and transcriptional differences between the parental strain and the strain evolved at low temperature.

## Results

### Competition and adaptive laboratory evolution at low temperature of a mixed culture of wine yeasts

**Fig. 1:**
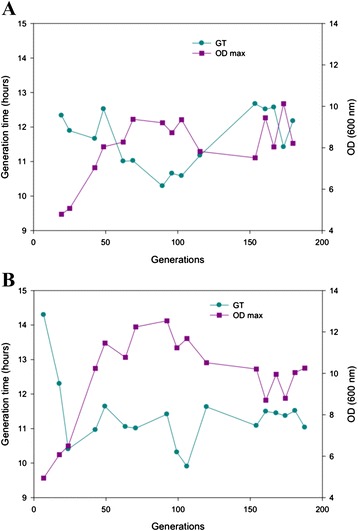
Growth evolution during competition and experimental evolution experiments. Evolution of growth (generation time and maximum OD_600_) in batch selection cultures in a synthetic must at 12 °C with no mutagenesis treatment (**a**) and with EMS mutagenesis treatment (**b**)

The growth of batch cultures at 12 °C was monitored during the whole competition and selection process (Fig. 1). The growth improvement of cultures was evidenced by the continuous increase of the maximum OD (OD_max_) and the reduction in the generation time (GT) throughout the first 100 generations. No clear growth improvements were observed between 100 and 200 generations, with fluctuations in the OD_max_ and GT values. In any case, the OD_max_ value after 200 generations of growth in SM at 12 °C was approximately 2-fold that of OD_max_ at the beginning of both the nonmutagenized and mutagenized cultures.

This growth improvement in the serial batch cultures can be explained firstly by the imposition of the most competent strain(s) of the mixed culture, and secondly by the evolution of these strains under selective cold pressure. Monitoring the 27 strains throughout the 200 generations confirmed the first hypothesis (Additional file 1: Figure S1). After 10 generations, only seven and nine strains were detected in the nonEMS and EMS cultures, respectively. Furthermore, major strain P5 already represented more than 50 % in both cultures. Only three strains were detected at generation 50, with P5 representing 57 % and 85 % in the nonmutagenized and mutagenized cultures, respectively. From generation 100 to 200, only strains P5 and P17 were detected in both cultures, and P5 was the major strain with percentages over 90 % at the end of the experiment.

### Fermentation performance of evolved strains in different synthetic and natural grape musts

The fermentation performance of the two strains isolated at the end of the serial batch cultures (P5 and P17) was analyzed. To distinguish between the strains isolated from the non-mutagenized or the mutagenized culture and the parental strains, the evolved strains were complemented with codes -E (evolved without EMS treatment) and EM (evolved and mutagenized), respectively. The fermentation kinetics of the original and evolved strains was estimated by calculating the time needed to ferment 5 % (T5), 50 % (T50) and 100 % (T100) of sugars in SM1 (Table 1) and is graphically plotted in Additional file 2: Figure S2. T5, T50 and T100 approximately match the beginning (lag phase), middle (end of exponential phase) and end of fermentation, respectively.

**Table 1:** Determination of the time required by the evolved and original strains to ferment 5 % (T5), 50 % (T50) and 100 % (T100) of the initial sugar content in a synthetic must (SM1) at 12 °C and 28 °C

Strain	P5	P5-E	P5-EM	P17	P17-E	P17-EM
**28 °C**						
T5 (h)	6.30 ± 0.74	5.98 ± 0.81	10.04 ± 0.81*	7.71 ± 0.90	9.96 ± 1.83	9.28 ± 0.34*
T50 (h)	38.54 ± 0.74	38.54 ± 2.25	37.7 ± 0.98	50.39 ± 2.05	48.93 ± 1.55	48.14 ± 0.90
T100 (h)	127.38 ± 3.43	126.31 ± 5.64	89.47 ± 11.84*	147.27 ± 3.98	125.29 ± 21.15	149.7 ± 0.00
**12 °C**						
T5 (h)	29.625 ± 3.25	20.81 ± 0.80*	22.88 ± 2.60*	65.74 ± 5.21	64.61 ± 4.27	54.09 ± 9.63
T50 (h)	211.88 ± 48.94	172.88 ± 47.43	104.63 ± 8.11*	335.83 ± 14.39	203.98 ± 9.82*	210.36 ± 9.04*
T100 (h)	-	505.69 ± 97.85*	349.13 ± 68.85*	-	539.43 ± 5.21*	570.1 ± 8.46*

*Statistically significant differences (*P*-value ≤ 0.05) compared with their control strain at the same temperature

- Unfinished fermentation

All the evolved strains showed better fermentation performance than the original strains at 12 °C (Table 1 and Additional file 2: Figure S2). The most remarkable improvement in the time to complete the low-temperature fermentation was observed in P5-EM. This strain took around 350 h to finish fermentation, whereas parental strain P5 was unable to consume all the sugars after 30 days (720 h) of fermentation. Strain P5-EM also reached a maximum OD that was 2-fold higher than original strain P5 (Additional file 2: Figure S2). The other evolved strains, P5-E, P17-E and P17-EM, exhibited similar fermentative behavior, and finished fermentations in more than 500 h. In any case, these three evolved strains also displayed improved fermentation performance and biomass production (higher OD yield) than the parental strains at low temperature (P17 was also unable to consume all the sugars after 30 days). Conversely, these differences in the growth rate and fermentation were absent or minimal during the fermentations at 28 °C.

In an attempt to take a step forward to approach wine industrial conditions, fermentations were carried out with the evolved strains in three natural grape musts (two white grape varieties, “Albariño” and “Macabeo”, and one red grape variety, “Garnacha”). Quite surprisingly, we did not observe any differences in fermentation performance between the evolved and parental strains under these conditions (data not shown). These results encouraged us to check the fermentation performance of evolved strain P5-EM at 12 °C in a new synthetic grape must (SM2), where YNB was replaced with a defined concentration of mineral salts and vitamins (Additional file 3: Figure S3A). As in the natural grape musts, no differences in both fermentation rate and biomass production were observed between the original and evolved strains.

### Determining the limiting nutrient at low temperature in SM1

**Fig. 2:**
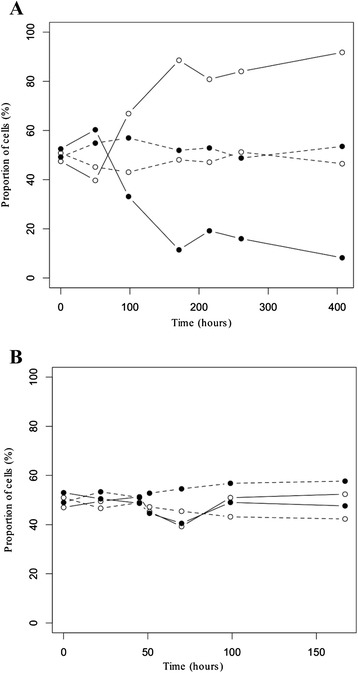
Population dynamics of P5 and P5-EM in SM1 and SM1 + I musts. Cell percentages of P5 (filled circles) and P5-EM (open circles) during the competition fermentations in SM1 (solid lines) and SM1 + inositol (20 mg/L) (dashed lines) at 12 °C (**a**) and 28 °C (**b**)

**Fig. 3:**
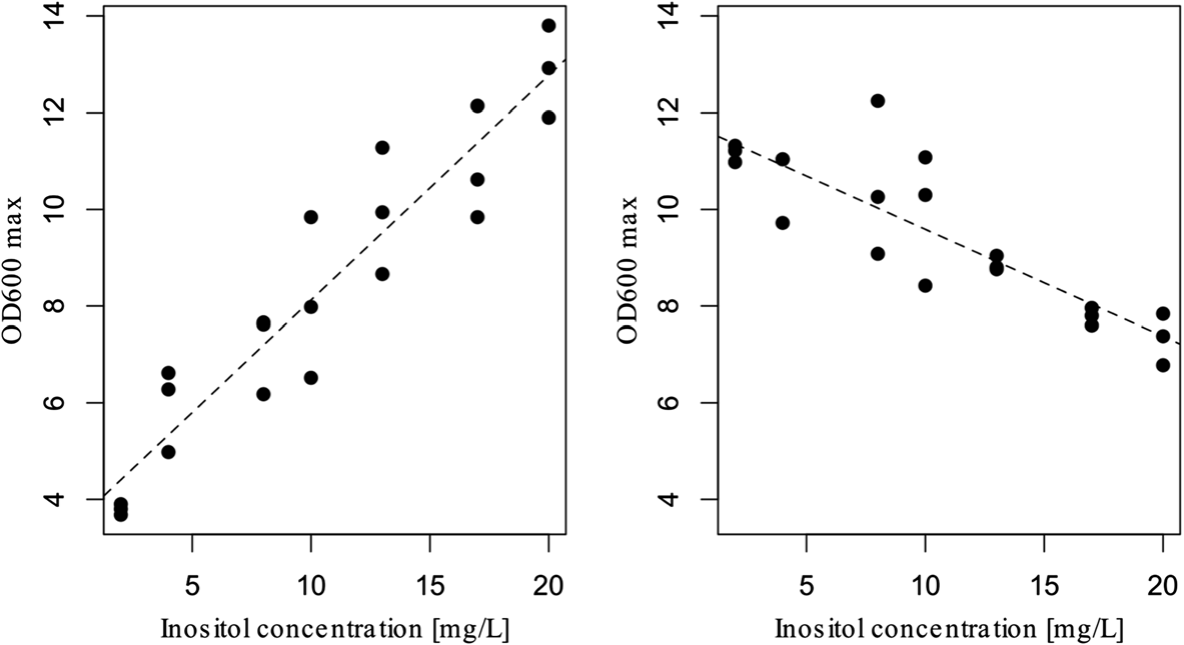
Relation between inositol concentration and maximum OD_600nm_. Maximum OD_600nm_ of P5 (left panel) and P5-EM (right panel) during the fermentations at 12 °C in SM1 and SM1 amended with different inositol concentrations. The line represents linear regression

The different fitness noted between the parental and evolved strains observed in SM1, but not in SM2, should be explained by the presence of a limiting nutrient (vitamin or mineral salt) or inhibitor (potassium disulfite) in this medium, and the evolution process had adapted strains to overcome this limitation/inhibition. It should be borne in mind that the evolution process was performed in the SM1 medium. In order to determine the limiting nutrient in SM1 at 12 °C, we carried out the fermentations of P5 and P5-EM using SM1 amended with every single compound that varied between both synthetic musts (Additional file 4: Table S1). The limiting nutrient was determined as inositol because we observed a recovered phenotype of the parental strain when inositol was added at the same concentration as in SM2 (Additional file 3: Figure S3B). Remarkably, addition of only inositol to SM1 resulted in the same fermentation and growth performance between both strains, and the fitness advantage of P5-EM disappeared (Fig. 2). Moreover, the OD_max_ at 12 °C of the parental strain correlated positively with an increasing inositol concentration (OLS regression slope: 0.465, R^2^: 0.893, P-value < 0.001), whereas the evolved strain OD_max_ correlated negatively with an increasing inositol concentration (OLS regression slope: −0.221, R^2^: 0.754, P-value < 0.001) (Fig. 3).

### The transcriptomic analysis of evolved strain P5-EM highlighted the strong up-regulation of mannoprotein genes

The global gene expression of P5-EM was analyzed during the SM1 fermentation at 12 °C and compared with its parental strain P5. The physiological condition chosen for analyzing the gene expression was the mid-exponential growth phase (96 h). The comparative results of the transcriptional profiles revealed that only 2.6 % (161/6124) of yeast genes showed significant differences in the transcript levels of at least 2-fold (Additional file 5: Table S2). Of these, 107 genes displayed a reduced expression in the evolved strain compared to the parental one, whereas only 54 genes exhibited an increased expression in P5-EM.

A MIPS categories analysis was done with the up- and down-regulated genes (Additional file 6: Table S3). The largest percentage of the up-regulated genes in P5-EM belonged to the functional category “metabolism” (40 %) and the sub-category “C-compound and carbohydrate metabolism” (22 %). Among the down-regulated genes, functional categories “transcription” and “protein with binding function or cofactor requirement” gave the largest percentages of genes (29.90 % and 24.29 %, respectively).

**Fig. 4:**
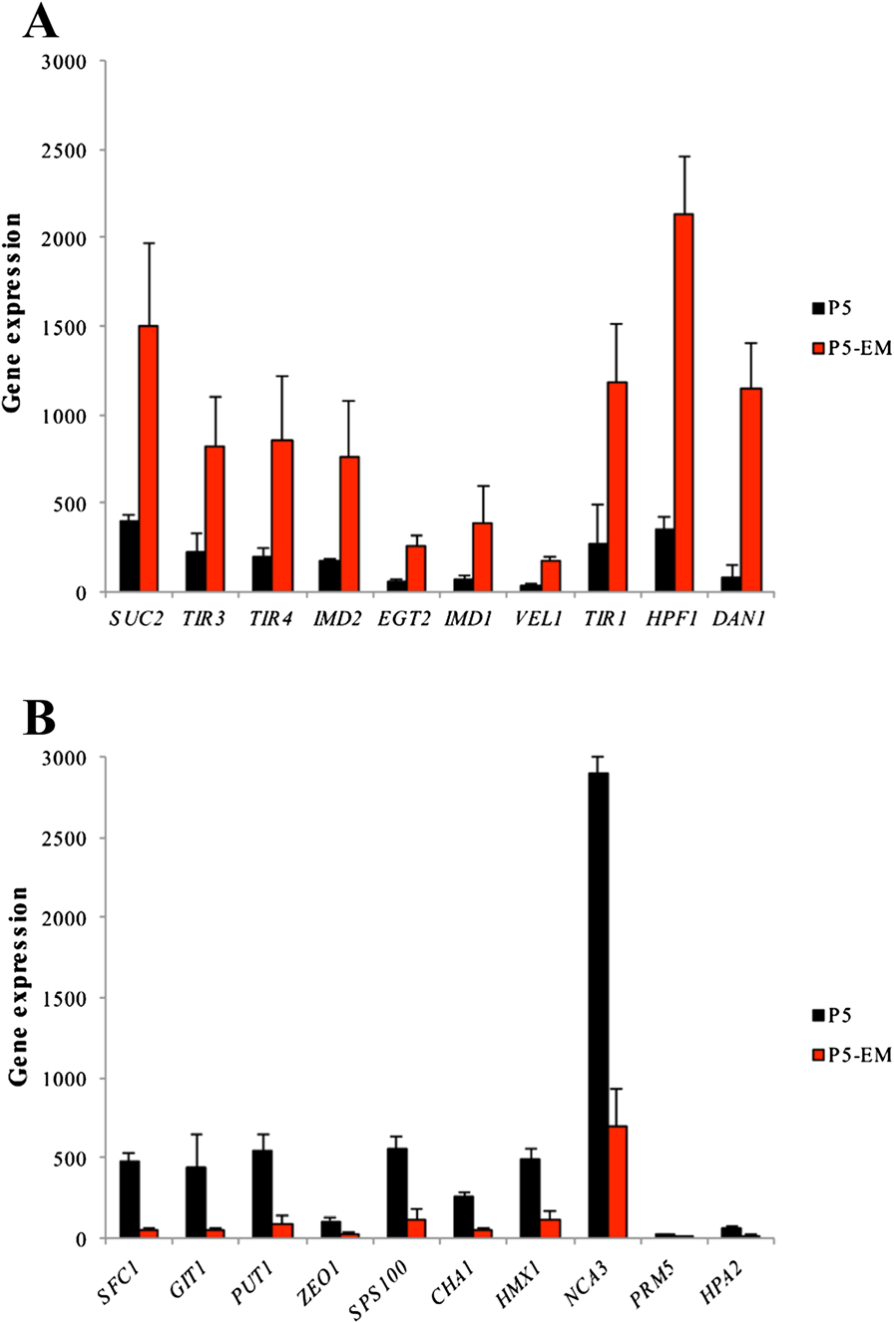
Major differences of transcriptomics analysis. The 10 most up-regulated (**a**) and down-regulated (**b**) genes in P5-EM (red bars) compared to P5 (black bars) during the fermentation at 12 °C

When we focused on the most strongly up-regulated genes in P5-EM (Fig. 4a), four belonged to the *DAN*/*TIR* family (*DAN1, TIR1, TIR4* and *TIR3*), which are cell wall mannoprotein genes, widely linked to a low temperature response [23]. *HPF1*, which encodes a mannoprotein that performs protective functions against protein aggregation in wines [24], also appeared among these top-ten up-regulated genes. The highest down-regulated genes (Fig. 4b) were: *SFC1* (encodes a mitochondrial succinate-fumarate transporter)*; GIT1* (encodes a plasma membrane permease that mediates the uptake of glycerophosphoinositol and glycerophosphatidylcholine); and *PUT1* (encodes proline oxidase).

### Metabolic changes in mannoprotein content and lipid composition

**Fig. 5:**
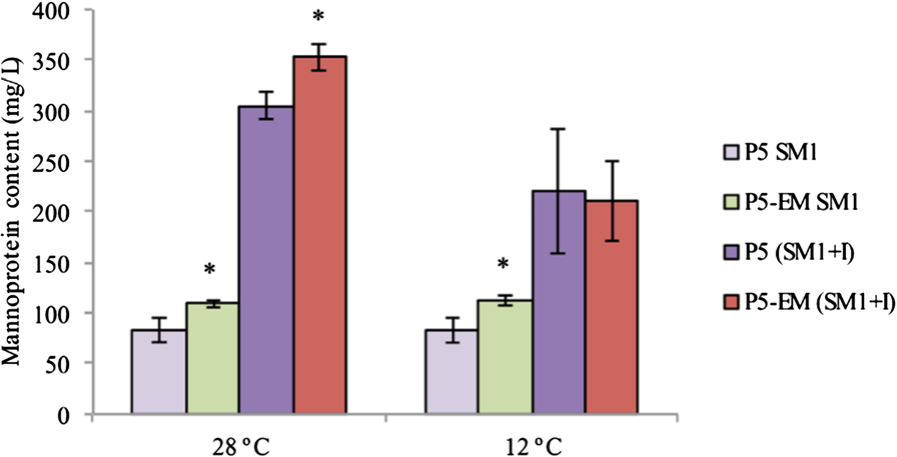
Mannoprotein content. Final concentrations of mannoprotein (mg/L) released by P5 and P5-EM during the fermentation at 28 °C and 12 °C in SM1 and SM1 + inositol (20 mg/L)

As the transcriptomic analysis revealed this strong up-regulation of several mannoprotein genes, the amount of extracellular and cellular mannoproteins was determined during the fermentations of SM1 and SM1 supplemented with inositol (SM1 + I) at 28 °C and 12 °C respectively (Fig. 5 and Table 2). Evolved strain P5-EM released more mannoproteins to the growth medium because the wines fermented with this strain presented higher mannoprotein content, regardless of temperature. However, the greatest increase in mannoprotein content occurred with the enrichment of synthetic grape must with inositol. The final SM1 + I wines had mannoprotein concentrations of around 2- and 3-fold more than the SM1 wines. It is noteworthy that the cellular mannoprotein content (Table 2) correlated negatively with content in wines (Fig. 5). The strains grown in the inositol-limited medium (SM1) obtained a higher cellular concentration than the cells grown in excess inositol (SM1 + I). Evolved strain P5-EM also showed higher cellular concentrations than parental strain P5.

**Table 2:** Mannoprotein content in cell wall yeast (mg mannoprotein/g dry weight (DW)) in the mid-exponential growth phase (24 h at 28 °C and 96 h at 12 °C) during fermentation in SM1 and SM1 + Inositol (20 mg/L)

Strain	P5	P5-EM
**28 °C (24 h)**		
SM1	192.68 ± 25.16	221.24 ± 16.79*
SM1+ I	145.87 ± 30.96	140.22 ± 13.63
**12 °C (96 h)**		
SM1	199.65 ± 8.76	264.68 ± 36.63*
SM1 + I	124.64 ± 9.10	158.23 ± 14.80*

**Fig. 6:**
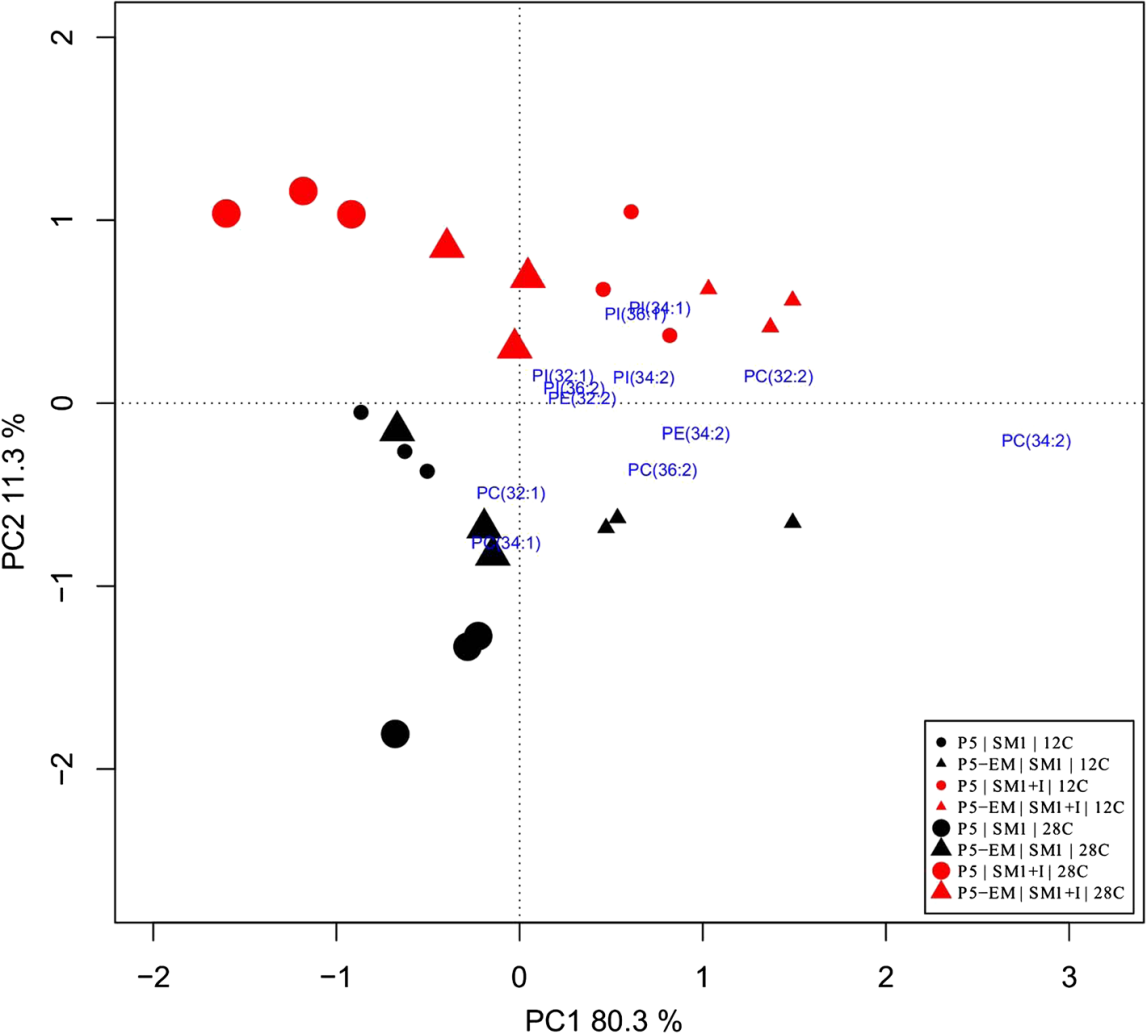
Principal component analysis of lipid composition. Biplot of the first two PCA components according to the lipid composition of strains P5 and P5-EM during the fermentations at 28 °C and 12 °C in SM1 and SM1 + inositol (20 mg/L)

Likewise, the phospholipid composition of the parental and evolved strains was also determined during fermentation in both media with limited and excess inositol. The percentage of the main phospholipid classes and their distribution in the different molecular species (phospholipid molecules varying in length and number of double bonds) are shown in Tables 3 and Additional file 7: Table S4, respectively. In order to highlight the most important changes between strains, temperature and fermentation medium according to their phospholipid composition, a Principal Component Analysis (PCA) was performed on the 24 individual samples obtained (2 strains x 2 temperatures x 2 SM x 3 triplicates) (Fig. 6). The two first components were retained and explained 91.6 % of total variance. The first component explained 80.3 % of variance and was marked by high positive component loadings for PC (34:2) (+0.77) and PC (32:2) (+0.38). The second component explained 11.3 % of variation and was marked by positive components loadings for PI (34:1) (+0.38) and PI (36:1) (+0.35), and by high negative loadings for PC (34:1) (−0.56) and PC (32:1) (−0.36). The general ordination of the samples by PCA showed the formation of two groups along the first axis: SM1, associated with high values of PC (34:1) and PC (32:1); SM1 + I, associated with high values of PI (34:1) and PI (36:1). Within the SM1 + I group, another separation was observed due to temperature. Low-temperature samples were associated with high values of PC (34:2) and PC (32:2). A similar pattern was observed within the SM1 group, but the P5 samples at 12 °C were grouped with the 28 °C samples.

**Table 3:** Percentage of phospholipids (LysoPC, PC, LysoPE, PE, PI, PS, PA and PG) expressed as the mean ± SEM (standard error of the mean) of total cellular concentration of these compounds. Cellular concentration of total phospholipid (Total PL) expressed as nmol/mg of dry weight. Significant differences (P ≤ 0.05), bold letters, was examined with *t*-test and was compared P5-EM with P5 strain in each condition

	SM1				SM1 + Inositol			
	28 °C		12 °C		28 °C		12 °C	
	P5	P5-EM	P5	P5-EM	P5	P5-EM	P5	P5-EM
Total LysoPC	**4.33 ± 0.65**	**3.50 ± 0.19**	3.67 ± 2.09	2.05 ± 0.31	**1.04 ± 0.45**	**2.85 ± 0.06**	1.79 ± 0.24	1.75 ± 0.30
Total PC	51.32 ± 1.59	49.82 ± 1.35	53.14 ± 1.72	53.64 ± 2.23	**32.35 ± 3.28**	**44.89 ± 3.09**	46.90 ± 3.44	49.44 ± 0.24
Total LysoPE	3.16 ± 0.46	2.17 ± 0.08	**1.71 ± 0.24**	**1.16 ± 0.09**	**0.98 ± 0.20**	**1.68 ± 0.03**	**1.23 ± 0.07**	**1.04 ± 0.08**
Total PE	**18.29 ± 0.14**	**16.15 ± 0.57**	**21.99 ± 0.85**	**18.81 ± 1.10**	15.12 ± 3.99	15.52 ± 0.15	**19.86 ± 0.17**	**16.75 ± 0.17**
Total PI	**9.49 ± 1.60**	**16.77 ± 1.16**	**8.36 ± 0.57**	**17.42 ± 3.28**	**40.42 ± 2.99**	**29.21 ± 3.70**	24.25 ± 4.47	27.11 ± 0.82
Total PS	5.21 ± 0.89	5.06 ± 0.47	2.66 ± 0.23	3.06 ± 0.36	7.49 ± 3.86	2.27 ± 0.92	**3.80 ± 0.78**	**1.82 ± 0.58**
Total PA	**6.70 ± 0.37**	**5.60 ± 0.27**	**5.91 ± 0.33**	**2.97 ± 1.00**	**1.64 ± 0.33**	**3.21 ± 0.50**	1.52 ± 0.11	1.65 ± 0.10
Total PG	1.49 ± 0.65	0.92 ± 0.07	**2.56 ± 0.16**	**0.88 ± 0.25**	0.96 ± 0.58	0.38 ± 0.05	**0.65 ± 0.10**	**0.44 ± 0.07**
Total PL	25,81 ± 1.00	23.36 ± 4.40	**15.75 ± 2.43**	**32.44 ± 6.37**	**7.98 ± 5.51**	**23.81 ± 3.51**	**28.55 ± 1.98**	**35.44 ± 2.32**

### Changes in the genome of evolved strain P5-EM in comparison to parental strain P5: reconstruction of mutation *GAA1*^*Thr108*^

The whole genome of evolved mutant P5-EM was sequenced and compared with that of parent strain P5. We identified 18 single nucleotide polymorphisms (SNPs) in the P5-EM genome. Eight of these SNPs were nonsynonymous in the coding regions of eight genes (Table 4). Only one SNP, found in the *ODC2* gene, involved a change in regulatory sequence 5′ upstream of the ORF.

**Table 4:** Nonsynonymous single nucleotide polymorphisms (SNPs) identified in the coding regions in strain P5-EM in comparison to the parental strain (P5)

Chr	Coordinate	Ref.	Mut.	Change	Het/Hom	Gene	Molecular function
IV	119953	G	A	S547F	het	*UFD2*	ubiquitin-ubiquitin ligase activity
IV	974831	C	T	E318K	het	*YAP6*	sequence-specific DNA binding RNA polymerase II transcription factor activity
IX	108277	C	T	G1654D	het	*TAO3*	component of the RAM signaling network (molecular_function unknown)
V	143758	C	T	H623Y	het	*MIT1*	transcriptional regulator of pseudohyphal growth (molecular_function unknown)
XII	316429	C	T	T108I	het	*GAA1*	contributes to GPI-anchor transamidase activity
XIII	257816	G	A	P201S	het	*GIS4*	CAAX box containing protein of unknown function
XIV	128913	C	T	D58N	hom	*SEC2*	guanyl-nucleotide exchange factor activity
XV	848746	C	T	T90I	het	*HUA2*	cytoplasmic protein of unknown function

Several of the above-mentioned results indicated that the SNP in *GAA1* might explain the phenotypic differences observed between P5-EM and P5. This gene encodes a subunit of the GPI-protein transamidase complex, required to attach glycosylphosphatidylinositol (GPI) to the proteins in the ER, a mechanism by which proteins are attached to the cell surface in all eukaryotic cells. To check the relevance of this mutation, we constructed a site-directed mutant in *GAA1* to *GAA1*^*Thr108*^ in a derivative haploid of parental strain P5. We analyzed the fermentation performance of P5 *GAA1*^*Thr108*^ at 12 °C and 28 °C, which was compared with derivative haploid P5.

**Fig. 7:**
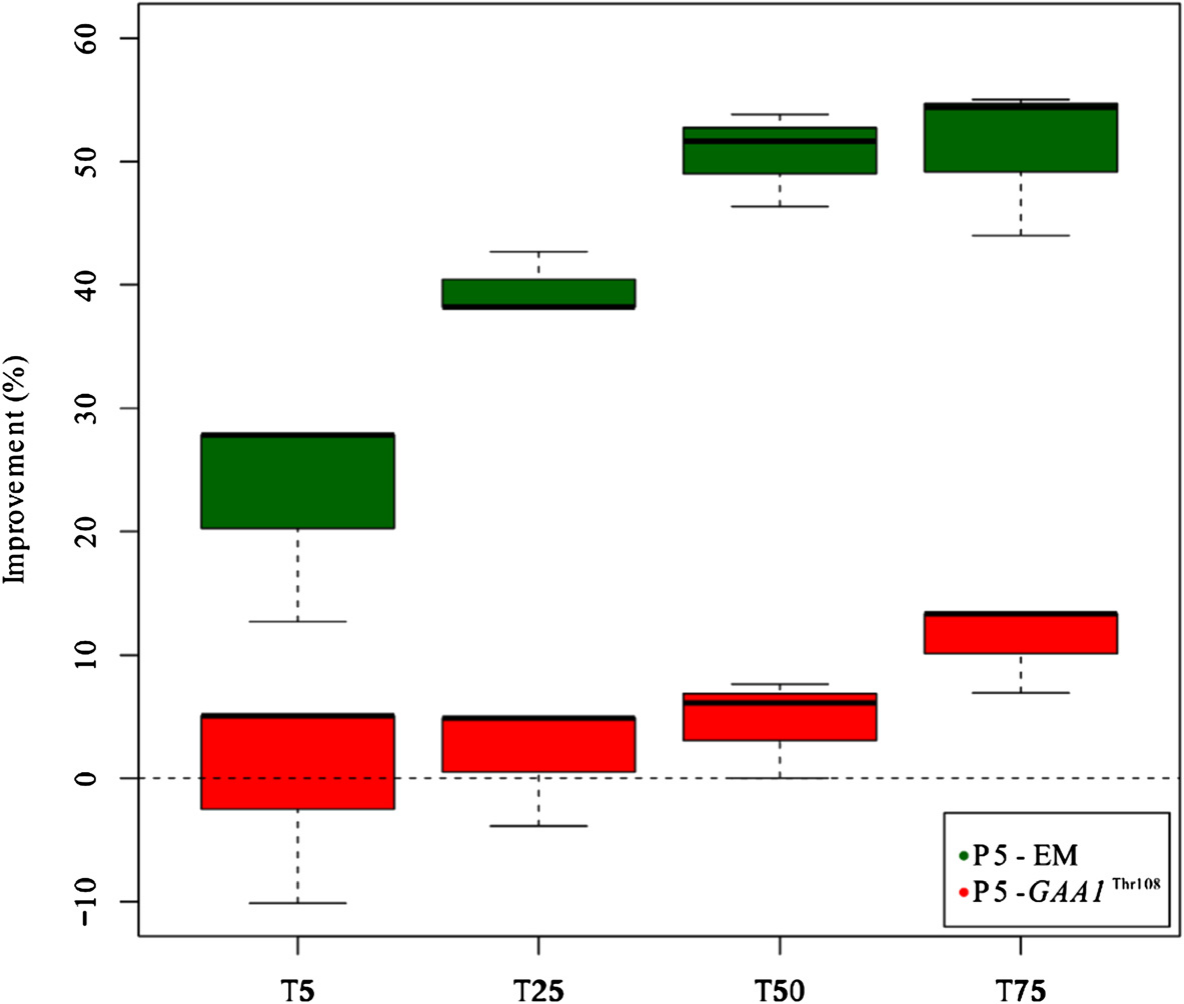
Dynamics of improvement in fermentation performance at 12 °C. Percentage of improvement in the fermentation kinetics at 12 °C (T5, T25, T50 and T75) of P5-EM and P5 *GAA1* ^*Thr108*^ compared to P5, and a derivative haploid of P5 *GAA1*, respectively

The fermentation kinetics of both strains was estimated by calculating the time needed to ferment 5 % (T5), 25 % (T25), 50 % (T50), 75 % (T75) and 100 % (T100) of the sugars in SM1. As previously observed for parental strain P5, its haploid was also unable to finish fermentation at 12 °C, whereas P5 *GAA1*^*Thr108*^ completed fermentation. The percentage of improvement at T5, T25, T50 and T75 during the fermentation at 12 °C of P5 *GAA1*^*Thr108*^ and P5-EM, in comparison to their respective parental strains, is plotted graphically in Figure 7. The results showed that P5-EM improved 50 % at T75, whereas the only mutation in *GAA1*^*Thr108*^ resulted in a 10 % improvement at T75, demonstrating that other mutations in addition to *GAA1*^*Thr108*^ are required to explain all of the improvement in fermentation in the evolved strain. At 28 °C, only strain P5-EM showed a slight improvement at T75 (Additional file 8: Figure S4). At the remaining time points analyzed at 28 °C, the parental strain mostly performed better than P5-EM and P5 *GAA1*^*Thr108*^.

## Discussion

In this study we used a batch serial dilution in a context that mimicked wine fermentation conditions at 12 °C to perform a competition experiment and experimental evolution. With this strategy, we attempted to select the most competent strains during the first generations, whereas longer exposure to low temperature led to an evolutionary adaptation of the genome of the previously selected strain/s. Monitoring strain diversity revealed that strain P5 was extremely competent very early in serial batch cultures. The mutational changes that led this strain to better adapt to low temperature fermentations were also denoted by improvements in growth parameters during the first 100 generations of the serial batch cultures. Subsequently, the fermentation test carried out in the same medium used for the evolution experiment confirmed the genetic improvement of this strain to grow and ferment at low temperature. However, it proved somewhat disappointing when we checked that this improvement in the growth and fermentation rates only happened in the context of the selection medium (SM1). We used YNB in this medium to provide the microelements (mineral salts and vitamins) needed for yeast growth and found the higher demand for inositol to grow at low temperature converted this vitamin into a growth-limiting substrate. Inositol is an essential phospholipid precursor in yeast cells and could be incorporated into phosphatidylinositol (PI), sphingolipids and glycosylphosphatidylinositol anchors [25]. It is known that phospholipid composition plays an essential role in adaptation to low-temperature fermentations [26]. Therefore, our results evidenced that evolved strain P5-EM has a fitness advantage in this inositol limitation context at low temperature. This advantage was lost in excess inositol or at an optimum temperature. Steensels *et al.* [27] previously stressed the importance of selection conditions matching the industrial parameters as closely as possible and, concerning to inositol content, SM1 did not show enough resemblance with natural grape must. In spite of this unexpected result, the strategy followed in this study provides evidence for the utility of competition experiments for detecting the most competitive strains among a pool of commercial strains, as well as evolutionary engineering as a nonrecombinant technique to isolate the commercial wine yeast strains that are highly tailored to stressful low-temperature wine fermentation conditions. In industrial settings, cells often face combinations of different stresses [28]. During wine fermentations, for example, cells encounter osmotic stress, high ethanol levels and nutrient deprivation. Through selection steps under conditions that resemble this harsh environment, researchers have succeeded in improving wine strains by evolutionary engineering [16–18].

Apart from the importance of obtaining improved strains to be used during wine fermentations in industry, another important aim of this study was to detect the physiological and molecular bases underlying this improved phenotype. For this purpose, metabolic, transcriptomic and genomic changes in evolved strain P5-EM were determined in comparison to parental strain P5. As mentioned above, our results clearly provide evidence for a higher demand for inositol when cells grew at low temperature. However the evolved strain was able to bypass this inositol requirement, to the extent that a negative correlation between inositol concentration and growth of this strain was observed. Wenger *et al.* [29] formerly reported that organisms adaptively evolved in limiting-nutrient environments perform worse than their ancestors in some cases when resources are nonlimiting. These trade-offs may result from either the adaptive mutations themselves or the accumulation of conditional loss-of-function mutations elsewhere in the genome.

Incubation at low temperature increases the molecular order of membrane lipids by rigidification [30]. Yeast responds to this rigidification by adapting different changes in the membrane lipid composition to maintain appropriate membrane fluidity. Nevertheless, inositol limitation denoted incapacity to reshape its lipid composition in the parental P5 strain. The inositol used in the synthesis of PI is either produced from glucose-6-phosphate or imported from the culture medium. Thus the fitness advantage observed in the evolved strain could potentially be explained by the optimization of the synthesis route to counteract the limitation of inositol in the culture medium. However we detected neither transcriptomic difference nor genomic changes in the genes involved in inositol biosynthesis or transport. The only likely exception was the strong down-regulation of *GIT1* observed in the evolved strain. This gene encodes a plasma membrane permease that mediates the uptake of glycerophosphoinositol and glycerophosphocholine as a source of inositol and phosphate [31, 32]. The lower inositol requirement of this strain could lead to down regulation of a gene involved in the transport of compounds that can be used as inositol sources. Yet, regardless of the direct involvement of inositol in the synthesis of PI, we should bear in mind its crucial role in the transcriptional regulation of many phospholipid biosynthesis genes [33]. The general impact of inositol on phospholipid biosynthesis was revealed by the differences found in the total phospholipid content in strain P5 growing at low temperature (almost 2-fold more in SM1 + I than in SM1; Table 3). Somehow the evolved strain is not subjected to this tight regulation by inositol because this strain showed similar phospholipid content when grown at low temperature in both media.

Another metabolic change in the evolved strain was the increase noted in extracellular and intracellular mannoprotein contents. These mannoproteins are attached to the cell wall via glycosilphosphatidylinositol (GPI) moieties, and are essential for cell wall integrity [34] and cell viability [35]. The GPI anchor is synthesized from PI through multiple steps in the ER and it is then transferred to the C terminus of proteins bearing a GPI attachment signal sequence. *GAA1* encodes a subunit of the GPI-protein transamidase complex, which is required for the attachment of a completed GPI anchor to proteins [36]. We have shown how the introduction of allele *GAA1*^*Thr108*^, detected in the evolved strain, into the parental strain represents a 10 % improvement in fermentation time. This single point-mutation can partially explain the improved fermentation behavior observed in the evolved strain at low temperature. Obviously, this SNP was unable to completely reproduce the fermentation improvement observed in the evolved strain, which indicates the existence of other genetic determinants of cold adaptation. The results presented herein provide valuable additional genes, which can be used as a starting point for future efforts to disentangle the genetic basis of low temperature adaptation.

Regardless of the possible connection between the deficient synthesis of GPI-anchored proteins and a lower fitness at low temperature, our results suggested the direct involvement of mannoproteins in cold adaptation. The overexpression of the mannoprotein genes in the evolved strain was related with increased mannoprotein content in the cell wall and an increase in the mannoprotein released at the end of fermentation. The induction of a subset of *DAN*/*TIR* genes has been previously related to hypoxia, high pressure and low temperature conditions [37, 23, 38]. In a recent work, we also detected that the overexpression of *TIP1* and *TIR2* improved fermentation activity and growth at 12 °C [39]. Abramova *et al.* [23] postulated that this adaptation event is related either to membrane fluidity and affects membrane properties, or, some of these proteins play a role in sterol transport.

Further indirect molecular evidence for the better fitness of the evolved strain at low temperature was the up-regulation of functional categories, such as “C-compound and carbohydrate metabolism” and “cytokinesis (cell division)/septum formation and hydrolysis”, which can explain the higher fermentation and growth rates observed in this strain. In order to find out other experimental situations that provoke a similar gene expression response to that obtained in this study, we used the SPELL tool via the *Saccharomyces* Genome Database (SGD) [40]. Interestingly, the datasets from published microarray experiments, whose expression profiles most closely resemble our data, dealt with the impact of mannose and inositol pyrophosphate on carbon utilization, protein glycosylation and environmental stress response [41, 42]. This result provided additional support for a correlation between transcriptomic data and inositol and mannoprotein metabolism.

## Conclusions

Our data provide clear evidence for the greater requirement for inositol for *S. cerevisiae* growth at low temperature. We evolved a commercial wine strain in an inositol-limiting environment at low temperature. Both the growth capacity and fermentation activity of this strain improved at low temperature, and showed clear physiological and molecular changes in comparison with its parental strain. The capacity to reshape its membrane lipid composition and a better mannoprotein synthesis capacity seemed to be paramount for this strain’s cold adaptation. The mutation observed in gene *GAA1*, involved in the synthesis of the GPI-anchored proteins, partially explained the improvement observed in the evolved strain. We also provide a short list of candidate genes for further exploration in the search for mechanisms involved in low-temperature adaptation in industrial yeasts.

## Methods

### Strains and mutagenesis

A pool of 27 commercial wine yeast strains was used in this study. The industrial strains were kindly provided by Lallemand Inc. (France) and were typed by their interdelta sequences [43], thus they were named according to their delta pattern (from P1 to P27). Their corresponding commercial names are shown in Additional file 9: Table S5 and their enological features can be obtained from the company’s website (http://www.lallemandwine.com). Before chemical mutagenesis, the yeast cells of each strain were grown overnight in 3 mL of YPD at 30 °C and 200 rpm. A mixed culture with the same proportion of each strain was prepared to a final cell concentration of 2 × 10^8^ cells/mL. This mixed culture was divided into two lots: one was mutagenized with EMS, following the protocol described by Winston [44], and the other was used as a nonmutagenized control. Both mutagenized and nonmutagenized cultures were further used as inocula of the competition and evolution experiments.

### Competition experiments and experimental evolution

After mutagenesis procedures, cells were transferred to a chemically defined synthetic grape must (SM1), recently described by Quirós *et al.* [45]. The SM1 composition included 200 g/L of sugars (100 g/L glucose + 100 g/L fructose), 6 g/L of malic acid, 6 g/L of citric acid, 1.7 g/L of YNB without ammonium and amino acids, anaerobic factors (15 mg/L ergosterol, 5 mg/L sodium oleate and 0.5 mL/ L tween 80) and 60 mg/L of potassium disulfite. The assimilable nitrogen source used was 300 mg N/L (120 mg N/L as ammonium and 180 mg N/L in an amino acid form).

The competition experiments and experimental evolution were based on batch serial dilution. Batch cultures were prepared in laboratory-scale fermenters using 100-mL bottles filled with 60 mL of SM1 fitted with closures that enabled carbon dioxide to escape and samples to be removed. The population inoculated in each flask was at an OD of approximately 0.2. Batch selection was performed at 12 °C, with continuous orbital shaking at 100 rpm for 200 generations.

Cultures were allowed to grow through a normal growth curve, with a weekly transfer of a small volume (the volume required to inoculate at an OD of 0.2) of the expanded culture into 60 mL of fresh medium. Batch cultures were plated on solid YPD at the initial point (0) and at 10, 50, 100, 150, and 200 generations, and 50 colonies of each sampling point were randomly selected and kept at −80 °C in 35 % (v/v) glycerol for further genotyping analyses.

Culture growth was monitored by measuring absorbance at 600 nm every 48 h. The number of generations was calculated by the equation: n = (log N_t_ - log N_0_)/log 2, where n is the number of generations, N_0_ is the initial OD and N_t_ is the OD at time t. Thus, the generation time (GT) was calculated by the equation GT = t/n.

### Interdelta sequences typing

Yeast typing was performed by delta element amplification from genomic DNA. PCR amplifications were carried out in a 50-μL reaction containing 5 μL (0.1-100 ng) of DNA, 1 μL of 200 μM dNTPs, 1 μL of 10 μM primers, 5 μL of 10 × PCR buffer, 2.5 μL of 50 mM MgCl_2_, 1 μL of BSA 200 μg/mL, 0.2 μL of Taq polymerase and 33.3 μL of water to a total volume of 50 μL. The delta sequence amplification conditions were those described by Legras and Karst [44]. Amplification products were separated by electrophoresis on 1.5 % (w/v) agarose gels.

### Strain construction

A site-directed mutant was constructed in point mutation *GAA1*^*Thr108*^. The *URA3* gene was replaced in strain P5 with the *KanMX4* gene following the SFH method [46]. After obtaining P5 (*ura3::KanMX4*), a derivative haploid of this strain was constructed by replacing the *HO* gene with the *HphMX4* (hygromycin resistance) cassette amplified from plasmid pAG32 [47]. Transformants were sporulated to select spores resistant to geneticin and hygromycin. The haploid state of the segregants was confirmed by PCR determination of the MAT locus [48]. After screening growth capacity at the low and optimum temperatures of the *HO* mutants, the haploid strain most like the parental wine strain was selected. This strain was then used to construct the site-directed mutant by cloning into the centromeric plasmid pGREG526, which contained *URA3* as a marker [49]. The *GAA1* gene was amplified from approximately 600 nucleotides upstream of the start codon and 400 nucleotides downstream of the stop codon to ensure that the promoter and terminator regions were included. The P5-EM (evolved) strain was used as a template to amplify the *GAA1*^*Thr108*^ allele. The plasmid was linearized by *Sal*I digestion and digested with *Not*I to avoid sticky ends and to make the recombination process easier. The selected haploid P5 (*ura3Δ*) strain was co-transformed with the digested pGREG526 plasmid together with the PCR-amplified *GAA1*^*Thr108*^ allele. This recombination between both fragments occurred *in vivo* and, during this process, the *GAL1* promoter of the plasmid was deleted. Thus the *GAA1*^*Thr108*^ allele was cloned with its own promoter. As control or reference strain, the haploid P5 (*ura3Δ*) strain was also co-transformed with the pGREG526 plasmid together with the wild-type *GAA1* allele. *Escherichia coli* strain DH5α was used for plasmid amplification. To check the selected clones, genes were sequenced and the presence of the site-directed mutation was confirmed.

### Fermentations

The fermentation experiments with the evolved strains were carried out in the same medium selected for competition experiments and experimental evolution (SM1) and in a more defined synthetic grape must medium (SM2), in which the YNB base was replaced with a defined concentration of mineral salts, as described by Riou *et al.* [50]; KH_2_PO_4_ 750 mg/L, K_2_SO_4_ 500 mg/L, MgSO_4_ 250 mg/L, CaCl_2_ 155 mg/L, NaCl 200 mg/L, MnSO_4_ 4 mg/L, ZnSO_4_ 4 mg/L, CuSO_4_ 1 mg/L, KI 1 mg/L, CoCl_2_ 0.4 mg/L, H_3_BO_3_ 1 mg/L and (NH4)_6_Mo_7_O_24_ 1 mg/L; and vitamins myo-inositol 20 mg/L, calcium pantothenate 1.5 mg/L, nicotinic acid 2 mg/L, chlorohydrate thiamine 0.25 mg/L, chlorohydrate pyridoxine 0.25 mg/L and biotin 0.003 mg/L. The assimilable nitrogen source used was 300 mg N/L (120 mg N/L as ammonium and 180 mg N/L in the amino acid form).

Natural grape must (NM) of two grape varieties (Albariño and Macabeo) and one red grape variety (Garnacha) were also fermented by wild strain P5 and their derivative evolved strains (P5-E and P5-EM). The three NMs contained approximately 200 g/L of reducing sugars (100 g/L glucose + 100 g/L fructose). Prior to inoculation, the natural grape must was treated with 1 ml/L of Velcorin (trade name for dimethyldicarbonate; Merck, Hohenbrunn, Germany). The use of this antimicrobial agent resulted in the practical elimination of the natural must microbiota, tested by plating on YPD and incubating for 72 h at 30 °C.

Fermentations were performed in laboratory-scale fermenters using 100-mL bottles filled with 60 mL of grape must and fitted with closures that enabled carbon dioxide to escape and samples to be removed. Fermentations were run at 28 °C and 12 °C with continuous orbital shaking at 100 rpm. The population inoculated in each flask was 2 × 10^6^ cells/mL from an overnight culture in YPD. Fermentations were monitored by measuring the density of the media (g/L) using a Densito 30 PX densitometer (Mettler Toledo, Switzerland). Fermentation was considered complete when density was below 998 g/L. Cell growth was determined by absorbance at 600 nm (OD_600_).

The fermentation kinetics was calculated by directly fitting density measurements versus time to the four-parameter logistic equation proposed by Speers *et al.* [51]. The estimation was done using the Sigmaplot software (Systa Software Inc. USA). When data were fitted to the four-parameter logistic equation, an estimation of time for each density value was also obtained. These values were used to calculate T5, T50 and T100. Fermentations were repeated at least 3 times, and data are reported as the mean value ± SD. Significant differences between strains were determined by *t*-tests (SPSS 13 software package, USA). The statistical level of significance was set at *P* ≤ 0.05.

### Determination of limiting nutrient during fermentation at low temperature

The main differences between synthetic grape musts SM1 and SM2 lay in vitamins, anaerobic factors and mineral salt concentration (Additional file 5: Table S2). To determine the limiting nutrient for growing in SM1 at 12 °C, each compound with a different concentration was added individually to SM1 at the same concentration as in SM2, whereas the rest of the compounds remained at the same concentration. Fermentations were carried out as described above.

To determine the limiting concentration of inositol, concentrations ranging from SM1 (2 mg/L) to SM2 (20 mg/L) were analyzed by adding aliquots to reach a final concentration of 2, 4, 8, 10, 13, 13, 17 and 20 mg/L. Fermentation was performed as described before. An ordinary least-square (OLS) regression was used to statistically test the relation between inositol concentration and OD_600_ max.

### Validation of the fitness advantage of evolved strain P5-EM

A GFP-labeled P5 strain (GFP- *KanMX4*) [52] was co-inoculated with evolved strain P5-EM in SM1 medium to compete during fermentation. The inoculated population was 2 × 10^6^ cells/mL (1 × 10^6^ cells/mL of each strain). The percentage of each strain throughout fermentation was monitored by both replica plating from YPD to YPD-geneticin (G-418, Formedium) and by flow cytometry. The percentage of fluorescent cells was determined in a flow cytometer (Beckman Coulter Epics XL Flow Cytometer, Minnesota, USA) after GFP induction in YP-Gal (1 % yeast extract, 2 % peptone and 2 % galactose) for 4 h at 25 °C (no changes in population size were detected during this incubation). In all, 20000 cells of the sample were measured at a voltage of 700 V in FL1 FITC, which revealed the number and percentage of fluorescent cells and fluorescence intensity. The EXPO 32 ADC software was used for these measurements. The parameters measured with the cytometer were number of fluorescent cells and average fluorescence intensity [53]. In order to rule out that the expression of GFP affects the fitness advantage of the parental P5 strain, a mixed inoculum of both strains (50 % P5-50 % P5-GFP) was inoculated in the same conditions explained above. The percentage of both strains was maintained at around 50 % throughout the fermentations, demonstrating that the fitness of the reporter strain did not diminish in comparison to that of the parental strain.

### Transcriptome analysis

Yeast cells (10^8^ cells/mL) were collected in the exponential growth phase during fermentation at 12 °C from three independently cultured replicates. RNA was isolated as described by Sierkstra *et al.* [54] and was re-suspended in 50 μL of DEPC-treated water. Total RNA suspensions were purified by the High Pure Isolation kit (Roche Applied Science, Germany) according to the manufacturer’s instructions. Solutions and equipment were treated so that they were RNase-free, as outlined in Sambrook *et al.* [55].

Microarray services were provided by the IRB Functional Genomics Core Facility, including quality control tests of total RNA by Agilent Bioanalyzer and Nanodrop spectrophotometry. RNA expression profiling was performed following the Pico Profiling method [56]. Briefly, cDNA library preparation and amplification were carried out from 25 ng total RNA using WTA2 (Sigma-Aldrich) with 17 amplification cycles. Eight μg of cDNA were subsequently fragmented by DNaseI and biotinylated by terminal transferase obtained from the GeneChip Mapping 250 K Nsp Assay Kit (Affymetrix). The hybridization mixture was prepared according to the Affymetrix protocol. Each sample was hybridized to a GeneChip Yeast Genome 2.0 Array (Affymetrix). Arrays were washed and stained in a Fluidics Station 450 and scanned in a GeneChip Scanner 3000 (both Affymetrix) according to the manufacturer’s recommendations. CEL files were generated from DAT files using the GCOS software (Affymetrix). To generate the log_2_ expression estimates, overall array intensity was normalized between arrays and the probe intensity of all probes in a probe set was summarized to a single value with the RMA (Robust Multichip Average) algorithm [57] in Genomics Suite 6.6 (Partek). Log_2_ ratios were used to calculate the differential expression between strains. The genes with at least 2-fold differences in the transcript levels (log_2_ratio was ≤ −1 or ≥ 1) between strains were tested. Genes were considered to have a significant differential expression if the p*-*values of the Student’s *t*-test were ≤0.05 after applying the Benjamini and Hochberg (BH) method to adjust for the false discovery rate (FDR) [58]. To group genes into functional categories, the GO term Finder in the MIPS Functional Catalog was used (http://mips.helmholtz-muenchen.de/funcatDB/).

### Illumina sequencing library prep

Illumina sequencing libraries were constructed from P5 and P5-EM. Genomic DNA was extracted with the Hoffman-Winston DNA preparation method. Bar-coded DNA fragment libraries were prepared by a Nextera DNA sample preparation kit (Epicentre Biotechnologies, Madison, WI) following standard procedures and published recommendations [59].

Briefly, 50 ng of yeast genomic DNA from each strain were tagmented (tagged and fragmented) by the Nextera transposome. The tagmented DNA was purified following the AMPure (Agencourt) purification protocol. Purified tagmented DNA libraries were PCR-amplified with the Nextera PCR Master Mix. PCR-amplified libraries were cleaned following the AMPure (Agencourt) purification procedures, and submitted for sequencing.

### Genome mapping and variant calling

First 591,334 paired-end, 100 bp, quality-filtered reads were collected from P5, and 1,793,478 from P5-EM, with the Illumina Hiseq 2000 platform. These strains were sequenced a second time and gave a final yield of 2,138,346 paired-end reads from P5 and 6,459,516 from P5-EM. Reads were aligned to the sacCer3 reference sequence using BWA [60] and default parameters for paired-end reads. Mapped reads were converted into a SAM file format for each strain. A file containing uniquely mapped reads was generated from the original SAM file to obtain a final percent coverage of 89.5 % and 93 % for P5 and P5-EM, respectively. A final filtered mpileup file was generated per strain using samtools [61] with a -C50 filter, as recommended by BWA.

For SNP calling, a filtered VCF file was generated using vcftools, generated from the filtered mpileup file after removing duplicate reads. The filtered VCF file contained 4,278 variant predictions from P5 and 8,212 from P5-EM. Additional filtering using in-house python scripts separated the variants that were identified only in the evolved strain, removed variants that were called in both the evolved and ancestral strain, and annotated the final variant call list [62]. Finally, 96 variants from P5-EM were manually examined in Integrative Genome Viewer [63] (IGV) for further prioritization. Variant predictions were validated by Sanger sequencing.

### Determination of mannoprotein content

The total mannoproteins released during fermentation was quantified at the end of the fermentative process. The relative mannoprotein content of the yeast cell wall was also determined. Yeast cells were collected in the exponential growth phase during fermentation at 28 °C and 12 °C, 24 h and 96 h respectively, from three independently cultured replicates.

The mannoprotein quantification method described by Quirós *et al.* [64] was used. Three mL of supernatant were gel-filtered through 30 × 10 mm Econo-Pac®10 DG disposable chromatography columns (Bio-Rad Laboratories, Hercules, CA). Cell and supernatant samples were subjected to acid hydrolysis and filtered through 0.22-μm pore size nylon filters (Micron Analitica, Spain). Then samples were analyzed by HPLC for the quantification of the glucose and mannose released during hydrolysis. Significant differences between the mannoprotein content of strain P5 and the evolved strain were determined by t-tests (SPSS 13 software package).

### Lipid composition analysis

Yeast cells (5–10 mg dry mass) were collected in the exponential growth phase during fermentation at 28 °C and 12 °C, 24 h and 96 h respectively. Prior to lipid extraction, a 100-μL solution of cold methanol and 20 μL of EDTA 0.1 mM were added to the yeast cells with 1 g glass beads (0.5 mm, Biospec Products, USA) in Eppendorf tubes, then mixed for 5 min in a mini-bead-beater-8 (Biospec Products, Qiagen, USA). Lipid extraction was performed according to the protocol described by Redón *et al.* [65]. A lipid analysis was done at the Kansas Lipidomics Research Center (KLRC). An automated electrospray ionization-tandem mass spectrometry (ESI-MS/MS) approach was used to analyze the lipid composition in these samples, and data acquisition and analyses were carried out as described previously in Friederichs *et al.* [66]. Precise amounts of internal standards, obtained and quantified as previously described by Welti *et al.* [67], were added in the following quantities: 0.3 nmol di12:0-PC, 0.3 nmol di24:1-PC, 0.3 nmol 13:0-lysoPC, 0.3 nmol 19:0-lysoPC, 0.3 nmol di12:0-PE, 0.3 nmol di23:0-PE, 0.3 nmol 14:0-lysoPE, 0.3 nmol 18:0-lysoPE, 0.3 nmol di14:0-PG, 0.3 nmol di20:0(phytanoyl)-PG, 0.3 nmol di14:0-PA, 0.3 nmol di20:0(phytanoyl)-PA, 0.2 nmol di14:0-PS, 0.2 nmol di20:0(phytanoyl)-PS, 0.46 nmol 16:0–18:0-PI, 0.33 nmol di18:0-PI, 3.1 nmol tri17:1 TAG, and 4.6 nmol di15:0 DAG. The sample and internal standard mixture were combined with solvents so that the chloroform/methanol/300 mM ammonium acetate ratio in water was 300/665/35, and the final volume was 1.4 mL.

Unfractionated lipid extracts were introduced by continuous infusion into the ESI source on a triple quadrupole MS (4000QTrap, Applied Biosystems). Samples were introduced using an autosampler (LC Mini PAL, CTC Analytics AG, Zwingen, Switzerland), fitted with the required injection loop for the acquisition time and presented to the ESI needle at 30 μL/min. The peaks corresponding to the target lipids were identified and molar amounts were calculated in comparison to the two internal standards in the same lipid class.

Phospholipid species were annotated as “Abbreviation of the lipid class” (number of C in the fatty acyl chains : number of double bounds in the fatty acyl chains). For example, PC (32:2) means Phosphatidylcholine with a total of 32 C and two double bounds in the fatty acids. A principal component analysis (PCA) was done with the lipid composition data.

### Availability of supporting data

The data set supporting the results of this article is available in the Gene Expression Omnibus (GEO) Database repository GSE67428 (http://www.ncbi.nlm.nih.gov/geo/query/acc.cgi?acc=GSE67428) and in the Sequence Read Archive (SRA) database repository PRJNA277378 (http://www.ncbi.nlm.nih.gov/bioproject/277378). The data set supporting the results of this article is included in the article (and its additional files).

## Additional files

Additional file 1: Figure S1. Strain’s dynamics during competition and experimental evolution experiments. Percentages of *S. cerevisiae* wine strains in batch selection cultures in generations 10, 50, 100, 150 and 200 with no mutagenesis treatment (A) and with EMS mutagenesis treatment (B). Additional file 2: Figure S2. Fermentation kinetics of original and evolved P5 and P17 strains. Fermentation kinetics (measured as density reduction; dashed lines) and growth (measured as OD_600_; solid lines) of evolved and parental strains: P5 strains at 12 °C (A.1) and 28 °C (B.1) and P17 strains at 12 °C (A.2) and 28 °C (B.2). Original strains are represented as filled circles; the strains from the nonEMS treated cultures are represented as open circles; the strains from the EMS-treated cultures are represented as filled triangles. Additional file 3: Figure S3. Fermentation kinetics of P5 and P5-EM in SM2 and SM1 + I. Fermentation kinetics (measured as density reduction; dashed lines) and growth (measured as OD_600_; solid lines) of P5 (filled circles) and P5-EM (open circles) during the fermentation at 12 °C in SM2 (A) and SM1 + inositol (20 mg/L) (B). Additional file 4: Table S1. Main nutritional differences between both synthetic grape musts SM1 and SM2. Additional file 5: Table S2. A significant different gene expression list, with at least 2-fold differences in the transcript levels (log2-fold were ≤ −1 or ≥ 1) between P5 and P5-EM during low-temperature fermentation. Additional file 6: Table S3. Significant up- and down-regulated GO terms in P5-EM during low-temperature fermentation compared with P5. Numbers of genes are between brackets. Additional file 7: Table S4. Percentage of phospholipid molecular species expressed as the mean ± SEM (standard error of the mean) of total cellular concentration of these compounds. Symbol (−) ≤ 0.01 %. Significant differences (P ≤ 0.05), bold letters, was examined with *t*-test and was compared P5-EM with P5 strain in each condition. Additional file 8: Figure S4. Dynamics of improvement in fermentation performance at 28 °C. Percentage of improvement in the fermentation kinetics at 28 °C (T5, T25, T50 and T75) of P5-EM and P5 *GAA1* ^*Thr108*^ compared to P5, and a derivative haploid of P5, respectively. Additional file 9: Table S5. Yeast strains used in this study.

## Abbreviations

PL: Phospholipid
LPC: Lysophosphatidylcholine
LPE: Lysophosphatidylethanolamine
PA: Phosphatidic Acid
PC: Phosphatidylcholine
PE: Phosphatidylethanolamine
PG: phosphatidylglycerol
PI: Phosphatidylinositol
PS: Phosphatidylserine
