# Supplementary figures and images for: Evolutionary engineering of a wine yeast strain revealed a key role of inositol and mannoprotein metabolism during low-temperature fermentation
Source: BMC Genomics. 2015 Jul 22;16(1):537. doi: 10.1186/s12864-015-1755-2 (PMC4509780; doi:10.1186/s12864-015-1755-2)

**A**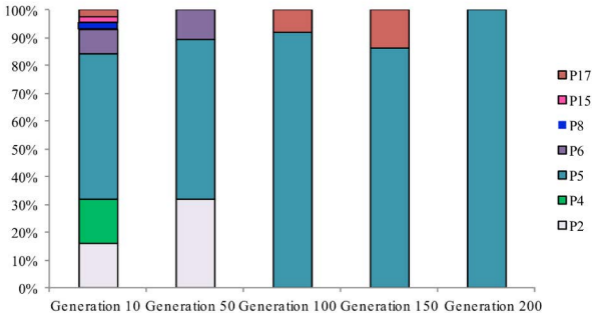**B**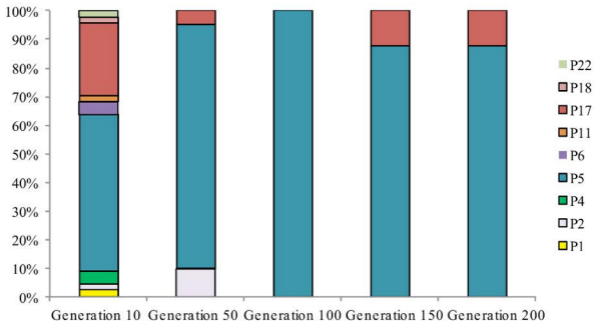

Supplement: Additional file 1: Figure S1. — Strain’s dynamics during competition and experimental evolution experiments. Percentages of S. cerevisiae wine strains in batch selection cultures in generations 10, 50, 100, 150 and 200 with no mutagenesis treatment (A) and with EMS mutagenesis treatment (B). [file 12864_2015_1755_MOESM1_ESM.pdf]

**A.1**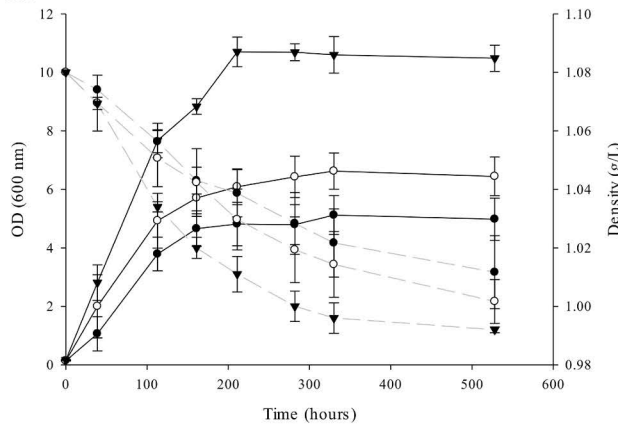**A.2**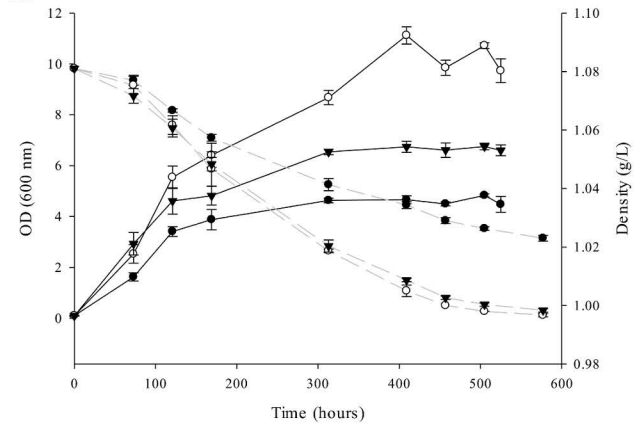**B.1**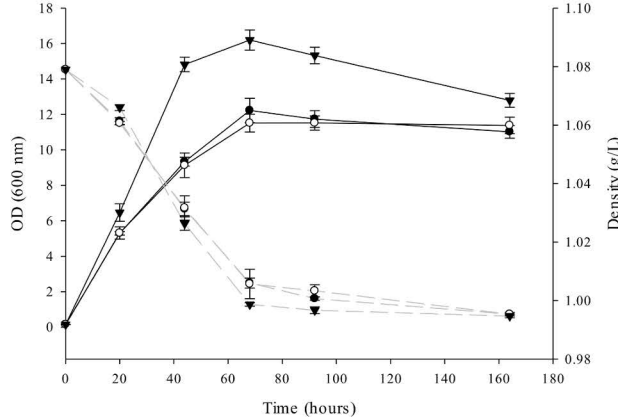**B.2**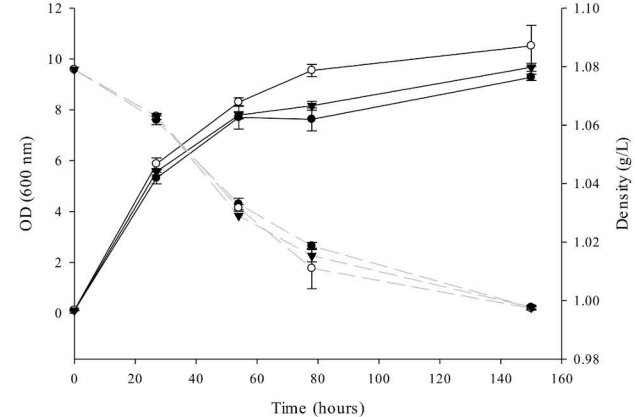

Supplement: Additional file 2: Figure S2. — Fermentation kinetics of original and evolved P5 and P17 strains. Fermentation kinetics (measured as density reduction; dashed lines) and growth (measured as OD600; solid lines) of evolved and parental strains: P5 strains at 12 °C (A.1) and 28 °C (B.1) and P17 strains at 12 °C (A.2) and 28 °C (B.2). Original strains are represented as filled circles; the strains from the nonEMS treated cultures are represented as open circles; the strains from the EMS-treated cultures are represented as filled triangles. [file 12864_2015_1755_MOESM2_ESM.pdf]

**A**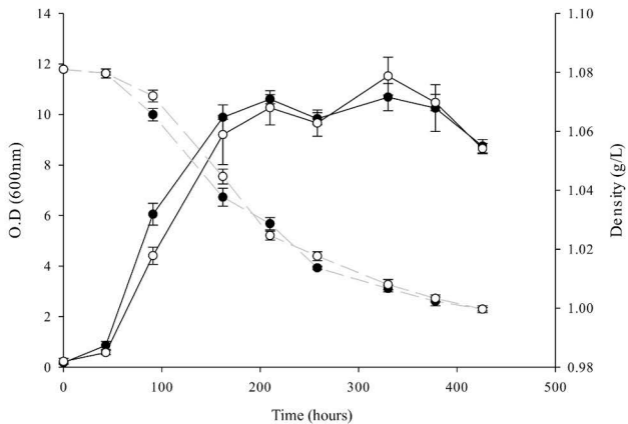**B**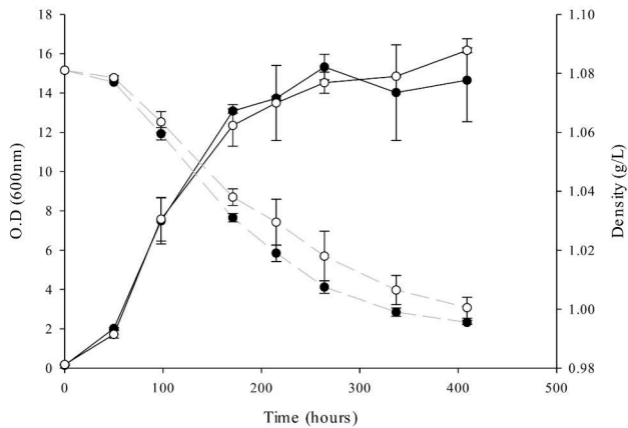

Supplement: Additional file 3: Figure S3. — Fermentation kinetics of P5 and P5-EM in SM2 and SM1 + I. Fermentation kinetics (measured as density reduction; dashed lines) and growth (measured as OD600; solid lines) of P5 (filled circles) and P5-EM (open circles) during the fermentation at 12 °C in SM2 (A) and SM1 + inositol (20 mg/L) (B). [file 12864_2015_1755_MOESM3_ESM.pdf]

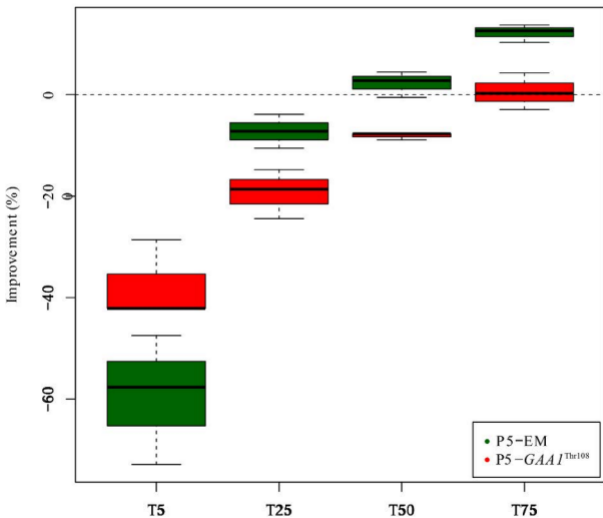

Supplement: Additional file 8: Figure S4. — Dynamics of improvement in fermentation performance at 28 °C. Percentage of improvement in the fermentation kinetics at 28 °C (T5, T25, T50 and T75) of P5-EM and P5 GAA1 Thr108 compared to P5, and a derivative haploid of P5, respectively. [file 12864_2015_1755_MOESM8_ESM.pdf]
